# Improvement to the Corrosion Resistance of Ti-Based Implants Using Hydrothermally Synthesized Nanostructured Anatase Coatings

**DOI:** 10.3390/ma7010180

**Published:** 2014-01-02

**Authors:** Martina Lorenzetti, Eva Pellicer, Jordi Sort, Maria Dolors Baró, Janez Kovač, Saša Novak, Spomenka Kobe

**Affiliations:** 1 Department for Nanostructured Materials, Jožef Stefan Institute, Jamova cesta 39, Ljubljana 1000, Slovenia; E-Mails: martina.lorenzetti@ijs.si (M.L.); janez.kovac@ijs.si (J.K.); spomenka.kobe@ijs.si (S.K.); 2 Jožef Stefan International Postgraduate School, Jamova cesta 39, Ljubljana 1000, Slovenia; 3 Departament de Física, Universitat Autònoma de Barcelona, Bellaterra E-08193, Spain; E-Mail: eva.pellicer@uab.cat (E.P.); dolors.baro@uab.es (M.D.B.); 4 Institució Catalana de Recerca i Estudis Avançats (ICREA) and Departament de Física, Universitat Autònoma de Barcelona, Bellaterra E-08193, Spain; E-Mail: jordi.sort@uab.cat

**Keywords:** titanium dioxide crystalline coating, corrosion resistance, nanomechanical behavior, biomaterials

## Abstract

The electrochemical behavior of polycrystalline TiO_2_ anatase coatings prepared by a one-step hydrothermal synthesis on commercially pure (CP) Ti grade 2 and a Ti13Nb13Zr alloy for bone implants was investigated in Hank’s solution at 37.5 °C. The aim was to verify to what extent the *in-situ*-grown anatase improved the behavior of the substrate in comparison to the bare substrates. Tafel-plot extrapolations from the potentiodynamic curves revealed a substantial improvement in the corrosion potentials for the anatase coatings. Moreover, the coatings grown on titanium also exhibited lower corrosion-current densities, indicating a longer survival of the implant. The results were explained by considering the effects of crystal morphology, coating thickness and porosity. Evidence for the existing porosity was obtained from corrosion and nano-indentation tests. The overall results indicated that the hydrothermally prepared anatase coatings, with the appropriate morphology and surface properties, have attractive prospects for use in medical devices, since better corrosion protection of the implant can be expected.

## 1. Introduction

A suitable combination of acceptable bulk mechanical properties and relatively good chemical stability makes titanium and its alloys the most used metals for body implants. The inertness of these materials is due to a biocompatible passivation layer of amorphous titanium dioxide, which is naturally formed on the surface [1,2]. Although this is considered as a protective interlayer between the hosting tissues and the foreign implant, it is too thin (only a few nanometres) and insufficiently stable under the action of body fluids to provide full corrosion protection, so that the bulk material may undergo a slow but lasting release of metal ions in the neighboring tissues. This may have an influence on defense mechanisms and cellular activity, and, as a consequence, adverse reactions and even implant rejection may occur [3,4]. For instance, vanadium, an alloying metal in Ti-Al-V alloys, has been found to be cytotoxic, producing harmful tissue effects [5–8], while aluminum has been suggested to be neurotoxic [9]. In addition, Ti-Al-V alloys are composed of a mixture of α (hexagonal close packed, HCP) and β (body-centered cubic, BCC) phases, resulting in a Young’s modulus that is typically much larger than that of bone, thus leading to undesirable stress-shielding effects and eventual loosening of the implant. To overcome this problem, metallurgical research has been directed towards the incorporation of “safer” alloying elements that promote the β-phase, which has a lower Young’s modulus than the α-phase. The use of the so-called β-stabilizer elements (Nb, Zr, Hf, Ta, Mo, *etc.*) has given rise to a new generation of Ti β-alloys.

In addition, it is known that the structure, physico-chemical composition and morphological characteristics of the biomaterial’s superficial zone, which is in direct contact with fluids and corporal tissues, determine the host biological response. Thus, surface optimization is important in order to minimize adverse body reactions and improve implant osseointegration; this is generally realized by modifying and increasing the stability of the natural titania passive layer. Surface modifications can be achieved by various techniques, according to mechanical (machining, grinding, polishing, *etc.*), chemical (e.g., etching, sol-gel deposition, *etc.*), electrochemical (anodic oxidation, micro-arc oxidation, *etc.*), physical (physical vapor deposition, thermal spray, *etc.*) or thermal (sintering, thermal oxidation, *etc.*) treatments [1,10,11]. Additionally, hydrothermal treatment (HT) emerged as a simple and cost-effective technique to produce thin, firmly attached layers of anatase with a well-defined morphology and crystallography, which offer the possibility to be used as a barrier to hinder the release of alloying elements and to enhance the bioactivity [12]. The influence of the surface porosity (or density) and the film thickness on these two aspects has to be further considered, in order to predict the longer-term corrosion effects as well as any possible bone reaction [13].

In our previous report on hydrothermally grown anatase crystals on titanium substrates [14], we have shown that such nanostructured coatings lead to a crystal morphology and surface topography that result in hydrophilic behavior. Moreover, high photocatalytic properties (proven by caffeine degradation and radical formation studies) and, accordingly, super-hydrophilicity upon UV-irradiation were observed. The combination of the two photo-induced phenomena may reduce the bacterial adhesion on the implant surface. Besides, in order to enhance the implant’s *in vivo* osseointegration, titania coatings are required to act as protective interlayers between the device and the surrounding tissues. Therefore, in the subsequent study the corrosion behavior of the anatase-coated Ti alloys was also examined. Relevant parameters related to the corrosion resistance were obtained by Tafel analyses of the corresponding potentiodynamic polarization curves. A correlation with surface morphology (e.g., porosity) was required to better interpret the corrosion data. In addition, nano-indentation of the selected anatase coatings was carried out to determine their hardness and elastic modulus.

## 2. Results and Discussion

The results are presented as a comparison of the properties within two groups of samples synthesized by hydrothermal treatment (HT) on commercially pure titanium (CP Ti) or a Ti13Nb13Zr alloy (TNZ), as highlighted in Table 1. The samples were hydrothermally treated under slightly different conditions to form different coatings: the samples Ti-A and Ti-B were prepared using different sources of titanium ions, while different times of HT were used in samples TNZ-C and TNZ-D. The non-treated substrates (Ti NT and TNZ NT) were used as the reference materials.

### 2.1. Morphological Characterization

**Figure 1.**
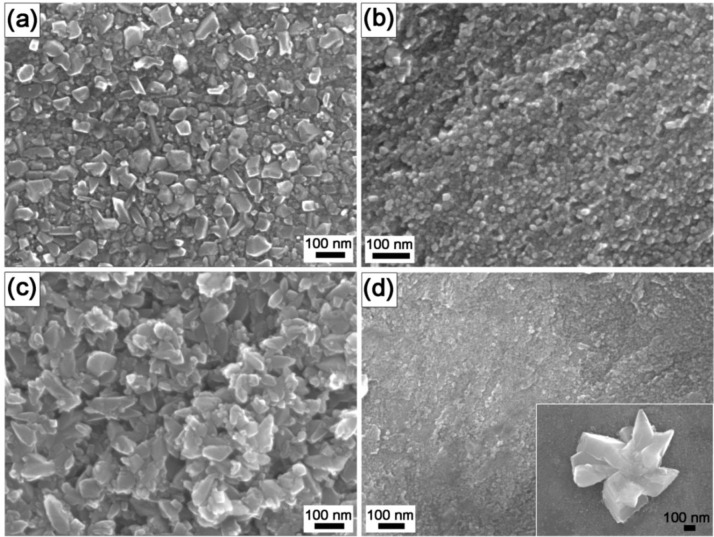
SEM micrographs of: (**a**) sample Ti-A; (**b**) sample Ti-B; (**c**) sample TNZ-C; and (**d**) sample TNZ-D.

As shown in field-emission-gun scanning electron microscope (FEG-SEM) images (Figure 1), the coatings completely covered the substrate surface; however, the crystals’ morphologies and dimensions were different. The coatings grown on the pure titanium substrates were composed of nanocrystals with dimensions ranging from 30 to 70 nm (Ti-A, Figure 1a) or 10 to 30 nm (Ti-B, Figure 1b). The same time of thermal treatment for the TNZ alloy (24 h) resulted in much larger (50–150 nm) TiO_2_ particles (TNZ-C, Figure 1c), while after only 12 h the estimated particle size was significantly smaller, 10–20 nm (TNZ-D, Figure 1d). In this case, a few large crystals, grown from the substrate with a flower/artichoke-like structure and randomly distributed, were also observed (inset in Figure 1d). Energy-dispersive X-ray spectroscopy (EDX) and X-ray photoelectron spectroscopy (XPS) analyses confirmed that the crystals composition was titanium dioxide. In the case of the TNZ samples, also ≈20 at.% of both zirconium oxide and niobium oxide were detected in addition to the TiO_2_ (data not shown). It was reported that from the electrochemical point of view, Ti- and Zr-rich regions are nobler than Nb-rich regions, giving more anodic protection [15], while Nb is able to stabilize the surface film, giving a higher cathodic protection [15] by filling the anion vacancies present on the titanium oxide layer [16]. Thus, the presence of zirconium and niobium oxides in the HT coatings is supposed to enhance the corrosion properties of the bare substrate.

Despite the variable morphology, the values of the surface mean roughness (*S_a_*) and the root-mean-square roughness (*S_q_*) obtained by atomic force microscopy (AFM) were not significantly different for all the samples (*S_a_* = 9.1 ± 0.7 nm and *S_q_* = 11.6 ± 1.0 nm) and, consequently, were considered negligible in this study. This decision was also supported by the findings of Aparicio *et al.* [17], who reported the ineffectiveness of the roughness on the qualitative electrochemical response of CP Ti and its alloys.

### 2.2. Electrochemical Corrosion Properties

During the stabilization time at the open-circuit potential (*E*_OCP_), the potential slightly shifted towards more positive values, indicating an increase in the surface oxide layer for the untreated specimens (Ti NT and TNZ NT) and the treated ones (with a minor variation). As shown by the *E*_OCP_ values listed in Table 2, the non-treated Ti had a slightly nobler value at the open-circuit potential (−0.429 V/SCE, saturated calomel electrode) in comparison with the TNZ (−0.647 V/SCE), indicating the tendency of the of the TNZ to spontaneously passivate in the Hank’s solution is less than for the pure Ti. Nevertheless, both values lay in the passivity region of the TiO_2_ at pH 7.4 in the Pourbaix diagram for titanium at 25 °C [18]. Even if a comparison with the literature data is not easy, due to its dependency on surface preparation, exposure time to the electrolyte and environment, potential scan rate, alloy composition and microstructure, *etc.* [19,20], the obtained *E*_OCP_ data were in agreement with previous reports [21–24]. All the hydrothermally treated (HT) samples showed less-negative *E*_OCP_ values, by about a factor of two, compared to the non-treated substrates Ti NT and TNZ NT. This confirms that the nanostructured titania coatings provided a more stable protection layer for the substrates in comparison with the natural passivation film present on the surfaces of the Ti alloys.

**Figure 2.**
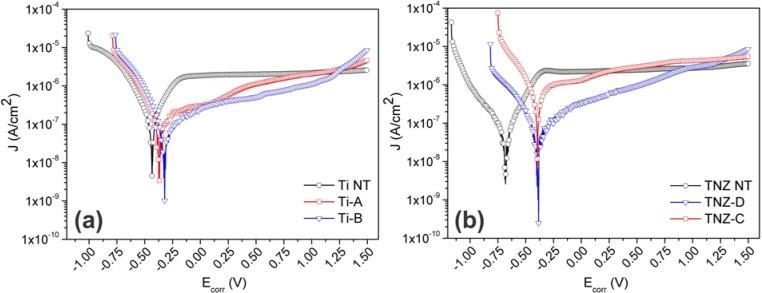
Potentiodynamic polarization curves in Hank’s solution at 37.5 °C of (**a)** samples Ti NT, Ti-A, Ti-B; and (**b**) samples TNZ NT, TNZ-C, TNZ-D.

Figure 2 illustrates the polarization curves on a logarithmic scale (Tafel plots) for the hydrothermally treated Ti and TNZ samples in comparison with the non-treated substrates. All the anodic polarization curves showed the same trend, typical for a passive character. The absence of a sharp increase in the current density in the anodic branch up to 1.5 V proved that no breakdown of the coatings occurred; however, current fluctuations near the cathodic-anodic transition point might depend on two events that occurred simultaneously: the tendency of current to increase because of the anodic reaction and the inhibition of the active surface of the electrode (thus decreasing in current) due to the oxide film’s formation [25]. In contrast to the curves for the bare substrates, the curves for the coated samples shifted towards the anodic region, qualitatively suggesting an improvement in the corrosion resistance.

According to Tafel’s law, the logarithm of the current density in an electrochemical reaction varies linearly with the electrode potentials (at potentials away from the OCP) [26]. Although not all the curves exhibit Tafel behavior (the anodic branch shows an active/passive transition), it is still possible to determine the anodic Tafel line from the experimental data, as shown by McCafferty for the Ti case [26]. The values of the corrosion potentials (*E*_corr_) and the corrosion current densities (*J*_corr_), calculated by the extrapolation of the cathodic and anodic branches of the curves at zero over-potential, are listed in Table 2. As expected, comparing the *E*_OCP_ and *E*_corr_ values for the Ti NT and TNZ NT, no appreciable difference was observed, meaning that the natural passivation layer was not able to grow and improve the surface corrosion properties. On the other hand, the *E*_corr_ of Ti-A and Ti-B slightly shifted with respect to their *E*_OCP_, suggesting that the coatings still had the ability to further passivate and stabilize, even after the 30 min of equilibration in OCP conditions. Moreover, considering the *E*_corr_ relative to Ti NT, a variation of ~0.10 V with respect to the positive potentials for the samples Ti-A and Ti-B was observed. Such a shift of the corrosion potentials indicated that the TiO_2_-coated surfaces possess a higher corrosion resistance. The same trend was found in the case of the TNZ samples, with an increase of the corrosion potential that is even higher (~0.30 V) compared to the non-coated substrate TNZ NT. The corrosion potential enhancement for the TNZ-coated samples was expected, as a result of the presence of highly protective zirconium and niobium oxides in the HT coatings. Furthermore, the *E*_corr_ values of the TNZ-C and TNZ-D were found to be similar to those for the Ti-A and Ti-B. This outcome demonstrated that the hydrothermal treatment is a simple but very powerful technique to grow very protective titania coatings, regardless of the substrate chemistry and composition.

Concerning the values of the corrosion-current density, a variation in *J*_corr_ of one order of magnitude was detected for the HT-treated Ti samples (~10^−8^ A/cm^2^) with respect to the Ti NT (~10^−7^ A/cm^2^). Such an outcome indicated that the rate of the oxidation process for the coated Ti substrates was reduced by the presence of the layer of titania nanocrystals, and as a consequence, the lifetime of the HT-coated titanium, under the applied conditions, was enhanced. The corrosion rate (CR) values are consistent with this trend (Table 2). Besides, no substantial enhancement in *J*_corr_ was noticed after the hydrothermal treatment of the TNZ samples (*J*_corr_ for TNZ NT in agreement with [27]). For the sample TNZ-C, the *J*_corr_ was even slightly worse compared to the TNZ NT. These results indicate that the TiO_2_-coating on the TNZ alloys delayed the onset of corrosion but did not slow down the corrosion rate with respect to the TiO_2_-coated titanium. As a result, further optimization of the synthesis parameters of the TNZ coatings series is required.

Although a lot of data on bare Ti and Ti13Nb13Zr can be found in the literature, information regarding the corrosion properties of synthesized TiO_2_ coatings is difficult to pin down. In fact, the variety of synthesis techniques, the different polarization parameters (e.g., scan rate, experimental time, potential, *etc.*) and the solutions used make any comparison very difficult. Nevertheless, a general consideration can be made.

Karpagavalli *et al.* [28] created an amorphous TiO_2_ layer on Ti6Al4V by electrodeposition and subsequent annealing. A variation of about +0.10 V in *E*_corr_ (comparable with the HT TiO_2_ coatings on Ti) was observed, while almost no difference in *J*_corr_ was obtained, compared the TiO_2_-deposited Ti6Al4V with Ti6Al4V in Hank’s solution. Similar variations in *E*_corr_ and *J*_corr_ were reported in [29], where Ti6Al4V substrates were spin-coated with TiO_2_ nanoparticles and then subjected to a further heat treatment. Indeed, an improvement of about +0.2 V in *E*_corr_ and of one or two orders of magnitude in *J*_corr_ was obtained, when comparing the results in the present study with the data obtained by Indira *et al.* [30], where the authors used anodized titanium sheets soaked in Hank’s solution for 1 h or 7 days. Furthermore, in Yu *et al.* [24], 400-nm anatase nanotubes were grown on Ti by anodisation and then crystallized by sintering. Equivalent corrosion values in Hank’s solution were obtained, proving that, besides more “conventional” methods for anodisation, a hydrothermal treatment is a powerful and very simple synthesis technique to obtain corrosion-resistant anatase coatings. Hydroxyapatite/titania (HA/TiO_2_) coatings were prepared using a hydrothermal-electrochemical co-deposited method by Xiao *et al.* [31]. They showed that such prepared composite coatings exhibited a better electrochemical behavior than pure HA coatings and uncoated Ti metal. Baszkiewicz *et al.* [32] also used a hydrothermal treatment on titanium as a second step after plasma electrolytic oxidation, in order to create titanium oxide layers enriched with HA in simulated body fluid (SBF). In this case the corrosion resistance of the HT layers was lower than that of the non-modified titanium. This outcome, combined with the considerations discussed within this section, indicated that the choice of synthesis parameters during the hydrothermal treatment is fundamental. Another advantage of the hydrothermally grown TiO_2_ coatings is their crystallinity. Park *et al.* [33] stated that in the case of a post-anodisation heat treatment, the crystallization of the TiO_2_ nanotubes on Ti rendered the layer very stable, showing the most effective corrosion resistance. In the case of the hydrothermal treatment, fully nanocrystalline coatings can be created in a one-step procedure, without any need for further post-treatment. To the best of our knowledge, we report for the first time the corrosion behavior, in a Hank’s balanced salt solution (HBSS), of polycrystalline anatase coatings prepared only by hydrothermal synthesis.

### 2.3. Thickness, Porosity and Nanomechanical Behavior

**Figure 3.**
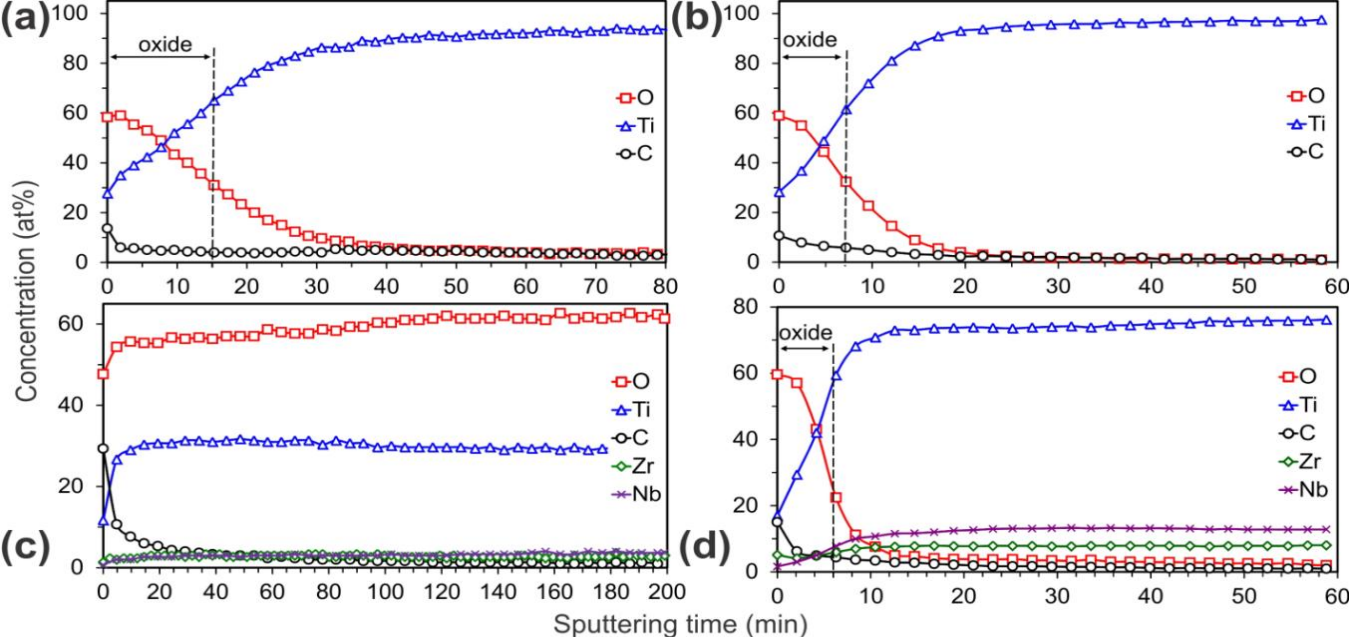
Auger electron spectroscopy (AES) profiles of the coatings on the samples: (**a**) Ti-A; (**b**) Ti-B; (**c**) TNZ-C; and (**d**) TNZ-D (etching rate: 2 nm/min). The thickness of the TiO_2_ anatase is indicated in each case.

Figure 3 shows the Auger electron spectroscopy (AES) depth profiles of the HT samples, which present the depth distribution of elements in the subsurface region. These profiles were obtained by ion etching with a rate of 2.0 nm/min. The oxide thickness was estimated from the etching time needed to reach the oxide/substrate interface. The oxide surface on all the samples was fairly rough, as can be seen in the SEM micrographs in Figure 1. This could strongly affect the quality of AES depth profiles. Due to the shadowing effect related to the surface roughness, the oxide crystals would not be etched uniformly by the ions, and therefore the oxygen signal in the AES profiles would persist for deeper regions than the real ones, giving an artefact signal. To avoid this, two ion guns from different directions were used to etch the titania crystals more uniformly. The oxide/substrate interface was estimated at the point where the oxygen concentration fell to half of its maximum value [34]. In all cases TiO_2_ was found to be formed at the surface. It appeared that for the TNZ-C (Figure 3c) the oxygen concentration remained as twice as high in comparison with the titanium for more than 200 min of etching time, indicating that the thickness of the TiO_2_ coating was greater than 400 nm. For this sample the oxide/substrate interface was not reached during the whole sputtering process. In contrast, for the other three examined samples (Figures 3a,b,d) the rapid oxygen decrease implies much thinner coatings. The estimated thicknesses are summarized in Table 3. From these results, the best corrosion resistance would be expected for the sample TNZ-C, due to the largest coating thickness, and, on the other hand, the smallest for the Ti-B (Figure 3b) and TNZ-D (Figure 3d). Unexpectedly, Ti-B and TNZ-D exhibited the best electrochemical behavior, despite their small thickness, an effect that could be connected with the compactness of the tiny anatase crystals that formed the coatings (Figures 1b,d, respectively). On the other hand, as mentioned above, the corrosion current density appeared worse in the case of the TNZ-C. This is evidence that the corrosion behavior cannot be fully related only to the coating thickness, but can also be influenced by the dissimilar porosity of the layers. For instance, even if the coating on sample TNZ-C is thick, the SEM image (Figure 1c) reveals its apparently high porosity. This feature possibly enlarges the exposed surface area to the HBSS (active anodic area), so that a consequent decrease in the total resistance would be expected [35]. In fact, although the corrosion potential shifted toward cathodic values with respect to the bare substrate, the corrosion rate was significantly higher. Furthermore, as suggested by Aparicio *et al.* [17], the increase of the current density might also be correlated with the surface compressive residual stresses, which in the case of sample TNZ-C may reside within the nanocrystals.

It is known that surface porosity has an effect on the corrosion behavior of alloys for biomedical applications [33] and that differences in *E*_r_ are dependent on the compactness of the coatings themselves [34,35]. According to the literature data [36,37], the Young’s modulus and the hardness (or compressive yield stress) can be correlated with porosity. Accordingly, indirect information about the porosity of the coatings was acquired in two different ways: (a) from the results of potentiodynamic analyses and (b) from the modulus of elasticity measured by nano-indentation, employing Equations (2) and (3), respectively (refer to experimental section).

**Figure 4.**
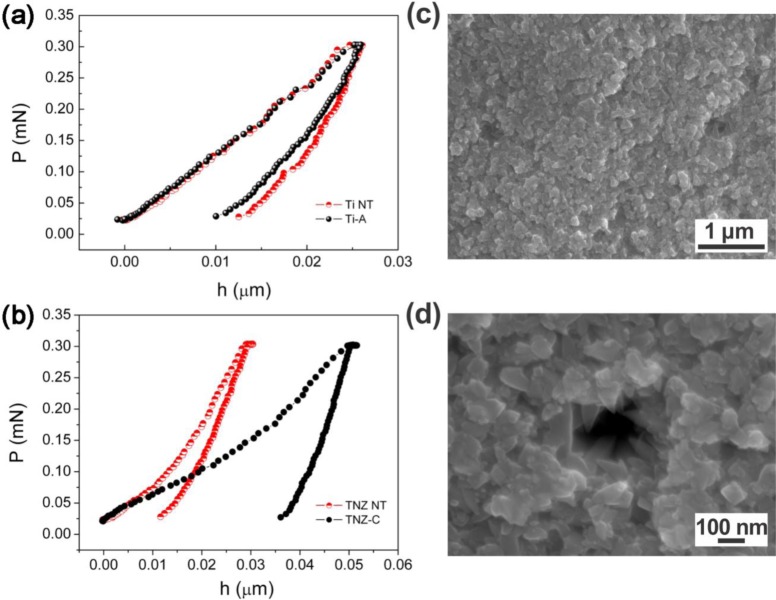
Representative load-displacement (P-h) nano-indentation curves (*P*_max_ = 0.3 mN) of: (**a**) samples Ti NT and Ti-A; (**b**) TNZ NT and TNZ-C; (**c**), (**d**) SEM images at different magnifications of the indent for sample TNZ-C.

The calculated values of the coating porosity from the potentiodynamic analyses are listed in Table 2. The trend of the values was in agreement with the visual feedback from Figure 1. Nano-indentation measurements on such prepared samples, and consequently calculations using Equation (3), were very challenging since several factors (*i.e.*, indentation size effect, surface roughness, nanostructured surface, corrective factors, *etc.*) need to be taken into account in order to avoid errors as much as possible and interpret the data correctly. So, although a rather small force was applied (0.3 mN), the displacement, or indentation depth, resulted in ≥20 nm in all samples (Figure 4). Bückle’s rule predicts no influence on the mechanical properties if the indentation depth is 1/10 of the overall film thickness. This implies that the measured mechanical properties in most of the coatings investigated here (in fact, all except the coating TNZ-C) were influenced, to a certain extent, by the contribution from the metallic bulk. The reduced Young’s modulus (*E*_r_) of the anatase coatings on the Ti appeared much more similar to the values reported in the literature for the anatase TiO_2_ (100–280 GPa) than for the ones reported on bulk rutile TiO_2_, which range between 340 and 380 GPa [34,38]. The *E*_r_ values are significantly higher than those reported for the non-treated annealed titanium metal (120 GPa) [39].

As expected, the hardness relative to Ti grade 2 (hexagonal close-packed α-phase) was higher than for Ti13Nb13Zr (body-centered cubic β-phase) (Table 3). This is due to the fact that the hexagonal cell is more anisotropic, and so less easily deformed in certain directions (reduced number of slip directions for dislocation motion). On the other hand, the more regular cubic structure possesses fewer constraints. As a result, the hexagonal structures (Ti samples) exhibited a higher hardness than the cubic ones (TNZ samples).

Comparing the H values of the bare substrates with the HT-treated ones, several conclusions can be drawn. The hardness was almost unchanged for the sample Ti-A with respect to the non-treated Ti (Ti-NT). Conversely, samples Ti-B and TNZ-D (which are the thinnest ones, corresponding to anatase with the smallest crystallite sizes) showed higher *H* values than the bulk metallic substrates. The reason for this could be two-fold: first, oxide materials are typically harder than metallic ones, due to their ceramic nature; additionally, grain-boundary strengthening, explained by the Hall-Petch relationship, could also play a crucial role in the observations [38]. In terms of dislocation motion, a material could be made infinitely strong if the grains are made infinitely small, so that the grain boundaries increase in number and hinder the propagation of dislocations, which become accumulated at the grain boundaries, thereby increasing the hardness.

Sample TNZ-C behaves differently. In this case, both *H* and *E*_r_ are much lower than for the non-coated TNZ (Table 3) and also much lower than for the bulk anatase. This has to be ascribed to the occurrence of porosity (*p*), as observed by FEG-SEM (Figure 1c) [39,40]. In principle, taking into account the thickness of the coating, nano-indentation data on the sample TNZ-C are more reliable (Figure 4b). Actually, the smoothness/linearity of the curves suggests that the loading-unloading process did not cause cracking [41]. However, due to the relatively large TiO_2_ particle size, interparticle voids inevitably occur within the coating, thus lowering *H* and *E*_r_ due to the resulting inherent porosity. The porosity in this sample can be estimated using Equation (3). If one takes the porosity value evaluated from the corrosion for sample TNZ-C (*p* = 27%), Equation (3) gives a value for the bulk Young’s modulus of 183.5 GPa, which falls well within the range of values for bulk anatase. Hence, the porosity assessments from the nano-indentation and corrosion measurements are consistent for this sample. For the other TiO_2_ coatings, which are very thin (Table 3), the porosity assessment from the nano-indentation curves does not make much sense since the *E*_r_ values are definitely influenced by the mechanical properties of the underlying metallic substrate. Namely, the reduction of *E*_r_ with respect to the bulk anatase can be both due to the influence of porosity and the lower Young’s modulus of the metallic alloy as compared to the bulk TiO_2_.

## 3. Experimental Section

Commercially pure titanium (CP Ti grade 2, ASTM F67, Pro-titanium, Baoji, China) and commercial Ti-β-alloy containing niobium and zirconium (Ti13Nb13Zr, ASTM F1713-08, Xi’an Saite Metal Materials Development Co., Xi’an, China) in the form of discs with a diameter of 15 mm, thickness of 2 mm, and grooves of width 0.03 mm after machining were used as the starting material for the hydrothermal synthesis. The substrates were cleaned twice in de-ionized water and once in absolute ethanol (EtOH, Carlo Erba, Milan, Italy) for 10 min each in an ultrasound bath (Sonorex, Bandelin Electronic, Berlin, Germany). The growth of the TiO_2_ anatase nanostructured films was performed by hydrothermal synthesis, using aqueous suspensions containing different titanium ions sources: one set of samples was prepared from aqueous suspensions of titanium dioxide anatase powder (μm-TiO_2_, 0.5 μm, Sigma-Aldrich Chemie GmbH, Munich, Germany) in different concentrations, and, eventually, ammonium citrate (AC, Kemika, Zagreb, Croatia) was added. Another series was synthetized according to a simple sol-gel route, where titanium(IV) isopropoxide [Ti(iOPr)_4_, Acros Organics, Geel, Belgium] was used as a TiO_2_ precursor in water. Moreover, the starting slightly acidic pH was adjusted for some suspensions by the addition of sodium hydroxide (NaOH, Kemika, Zagreb, Croatia) and/or tetramethylammonium hydroxide (TMAH, Sigma-Aldrich Chemie GmbH, Munich, Germany), reaching a pH of around 10. Teflon vessels, half-filled with the suspensions and containing also the bare substrates, were placed in steel autoclaves and heated up to 24 h at 200 °C (APT. line, Binder GmbH, Tuttlingen, Germany). After cooling to room temperature the samples were cleaned like before the HT treatment and dried in air. The samples’ preparation conditions are summarized in Table 1. The surfaces of the non-treated titanium (Ti NT) and Ti13Nb13Zr (TNZ NT) alloys were polished to a mirror-like finish before the corrosion and nano-indentation experiments and used as references. No surface polishing was applied to the HT-treated samples.

A field-emission-gun scanning electron microscope (FEG-SEM, Zeiss SUPRA 35VP, Carl Zeiss SMT, Germany and JEOL JSM 7600F, Tokyo, Japan) equipped with an energy-dispersive X-ray spectrometer (EDX) was used to observe the crystal morphology and to provide a rough estimate of the crystal dimensions. The chemical composition was estimated with the EDX, while the coating thickness was indirectly calculated from the profiles obtained using an Auger electron spectrometer (AES, PHI SAM 545 spectrometer). For the electron excitation a primary electron beam of 3 keV and 1 μA, with a diameter of 40 μm, was used. During the depth profiling the samples were etched by two symmetrically inclined Ar ion beams of 1 keV. The etching rate was measured on a reference Ni/Cr multilayer of known thickness and it was found to be 2.0 nm/min. The concentration of the elements was calculated from the corresponding signals in the AES spectra using the relative sensitivity factors provided by the instrument producer [42]. The surface mean roughness (*S*_a_) and the root-mean-square roughness (*S*_q_) were examined with an atomic force microscope (AFM, DiDimension 3100, Veeco Instruments Inc., Santa Barbara, CA, USA) on 1 × 1 μm^2^ areas.

Potentiodynamic polarization curves were recorded using a three-electrode cell configuration, with a platinum sheet as the counter electrode and a calomel reference electrode [SCE, +0.244 *vs.* normal hydrogen electrode (NHE)]. The samples were wrapped together with the electrode connection and then inserted, one by one, into the cell containing fresh Hank’s balanced salt solution (HBSS, Sigma-Aldrich). The solution was de-aerated with Argon flux and kept at 37.5 °C. The open-circuit potential (*E*_OCP_) *versus* time was recorded for 1800 s; after this period the system was considered stable. An autolab potentiostat instrument (PGSTAT 302N, Metrohm Autolab, Utrecht, Netherlands) was used for recording the potentiodynamic curves. In a typical experiment, the potential was swept from (*E*_OCP_ −0.5) V to (*E*_OCP_ +1.5) V at a scan rate of 0.5 mV/s. A Tafel plot extrapolation was carried out to calculate both the corrosion potential (*E*_corr_) and the corrosion-current density (*J*_corr_) values. For each sample, measurements were repeated at least twice. The corrosion rate (CR) was calculated as:

$$\mathrm{CR}=\frac{J_{\text{corr}}\cdot K\cdot EW}{d}$$ (1)

where *K* is a constant that defines the units of the corrosion rate [3.272 mm/(A cm year)], *EW* is the equivalent weight (11.98 g/eq for Ti alloys) and *d* is the density (4.51 g/cm^3^ for CP Ti and 5.01 g/cm^3^ for Ti13Nb13Zr [43]).

The mechanical properties of the thicker coatings were evaluated by nano-indentation (UMIS, Fischer-Cripps Laboratories, Forestville, Australia) using a Berkovich pyramid-shaped diamond tip and operating in the load control mode (30 s loading/20 s load holding/30 s unloading). The maximum applied force was 0.3 mN in order to minimize the influence of the underlying substrate. The thermal drift and compliance were automatically corrected by the software. The hardness (*H*) and the reduced Young’s modulus (*E*_r_) were evaluated using the method of Oliver and Pharr [44]; the presented results correspond to an average of a minimum of 50 indents.

Furthermore, surface porosity fraction was estimated by both potentiodynamic polarization and nano-indentation measurements. In the first case the porosity (*p*) can be calculated using the following equation:

$$p=\frac{R_{P,S}}{R_{P}}10^{-\left(\frac{\Delta E_{\mathrm{corr}}}{\beta_{a}}\right)}$$ (2)

where *R*_P,S_ and *R*_P_ are the polarization resistances of the bare substrate and the coating/substrate pair, respectively, Δ*E*_corr_ is the potential difference between them, and β_a_ is the anodic Tafel coefficient of the substrate.

In addition, Ramakrishan and Arunachalam’s approach was used for the porosity calculations from the nano-indentation measurements, based on the theory that the Young’s modulus is influenced by the interaction between the bulk solid material and the surface pores [45]. Thus, the porosity fraction can be extrapolated from the following equation:

$$\frac{E_{\mathrm{porous}}}{E_{\mathrm{bulk}}}=\frac{{\left(1-p\right)}^{2}}{1+2p-3\nu p}$$ (3)

where *E*_porous_ is the Young’s modulus of the porous material, *E*_bulk_ is the Young’s modulus of dense anatase and ν is the Poisson’s ratio of the bulk material (anatase in our case), which corresponds to 0.28, using literature data [46]. The Young’s modulus of the bulk anatase depends on the crystallographic direction, but it has been reported to range between 100 and 280 GPa [37,46].

## 4. Conclusions

This study concerned the electrochemical properties in Hank’s solution of polycrystalline anatase-TiO_2_ coatings prepared by hydrothermal treatment on two different alloys used for bone implants, *i.e.*, CP Ti grade 2 and Ti13Nb13Zr (TNZ). Tafel-plot extrapolations from the potentiodynamic curves showed a significant enhancement in the corrosion potentials for the anatase coatings, in comparison with the bare Ti and TNZ substrates (~0.10 V and ~0.30 V towards positive potentials, respectively). Moreover, the HT-Ti also exhibited lower corrosion-current densities and corrosion rates, indicating a longer implant lifetime. These results were correlated with the effects of the crystal morphology, coating thickness and porosity. Moreover, a general increase in the surface hardness and the Young’s modulus was observed for the coatings, due to the harder nature of the ceramic anatase, as compared to the Ti-based metallic alloys. However, the small thickness of the coatings made the measurements using the nano-indentation technique particularly challenging. To sum up, hydrothermally grown anatase coatings conferred improved corrosion resistance on the bare substrates, thanks to an appropriate morphology, coating thickness and porosity. Such coatings are expected to act as an efficient protective interlayer between the implant and the surrounding tissues and, consequently, a prolongation of the device’s lifetime *in vivo* is expected.

## Figures and Tables

**Table 1.** Summary of the synthesis conditions and information about the titania grown crystals for samples Ti-A, Ti-B TNZ-C and TNZ-D.

Sample	Substrate	Ti Ions Source	Additives	HT Time	Estimated Crystal Size after HT
Ti-A	CP Ti	Ti(iOPr)_4_	–	24 h	30–70 nm
Ti-B	CP Ti	μm-TiO_2_	AC, NaOH, TMAH	24 h	10–20 nm
TNZ-C	TNZ	Ti(iOPr)_4_	AC, NaOH, TMAH	24 h	50–150 nm
TNZ-D	TNZ	Ti(iOPr)_4_	AC, NaOH, TMAH	12 h	10–20 nm

**Table 2.** Open-circuit potential (*E*_OCP_), corrosion potential (*E*_corr_), corrosion current density (*J*_corr_), corrosion rate (CR) and coating porosity extrapolated from the potentiodynamic polarization curves. The errors in the *E*_OCP_ and *E*_corr_ values are within ±0.005 V, whereas for *J*_corr_ the value is typically around 0.1 × 10^−8^ A/cm^2^.

Sample	*E*_OCP_ (V/SCE)	*E*_corr_ (V/SCE)	*J*_corr_ (A/cm^2^)	CR (×10^−6^, mmpy)	Coating Porosity
**Ti NT**	−0.429	−0.442	1.84 × 10^−7^	1.599	–
Ti-A	−0.288	−0.359	6.92 × 10^−8^	0.601	17%
Ti-B	−0.262	−0.303	6.52 × 10^−8^	0.568	7%
**TNZ NT**	−0.647	−0.683	6.63 × 10^−8^	0.510	–
TNZ-C	−0.250	−0.386	3.27 × 10^−7^	2.513	27%
TNZ-D	−0.317	−0.396	4.77 × 10^−8^	0.367	2%

**Table 3.** Coatings thickness as obtained from AES; hardness (*H*) and reduced modulus of elasticity (*E*_r_) as obtained from nano-indentation measurements.

SAMPLE	Thickness (nm)	*H* (GPa)	*E*_r_ (GPa)
Anatase-TiO_2_ bulk	–	11.6 [36]	140 [36] (100–280 [37])
Ti NT	–	10.7 ± 0.2	126.8 ± 1.9
Ti-A	30 ± 6	10.8 ± 0.3	204.9 ± 4.9
Ti-B	14 ± 3	14.5 ± 0.3	171.9 ± 2.6
TNZ NT	–	7.6 ± 0.1	109.5 ± 1.1
TNZ-C	> 400	2.8 ± 0.1	74.6 ± 1.4
TNZ-D	12 ± 3	8.3 ± 0.1	106.2 ± 2.0
